# Abnormal Visual Scanning of Emotionally Evocative Natural Scenes in Huntington’s Disease

**DOI:** 10.3389/fpsyg.2017.00405

**Published:** 2017-03-29

**Authors:** Catarina C. Kordsachia, Izelle Labuschagne, Julie C. Stout

**Affiliations:** ^1^Monash Institute of Cognitive and Clinical Neurosciences, School of Psychological Sciences, Monash University,Melbourne, VIC, Australia; ^2^Cognition and Emotion Research Centre, School of Psychology, Australian Catholic University,Fitzroy, VIC, Australia

**Keywords:** Huntington’s disease, eye-tracking, emotion processing, emotional experience, visual scanning

## Abstract

Huntington’s disease (HD) is a neurodegenerative movement disorder associated with deficits in the processing of emotional stimuli, including alterations in the self-reported subjective experience of emotion when presented with pictures of emotional scenes. The aim of this study was to determine whether individuals with HD, compared to unaffected controls, display abnormal visual scanning of emotionally evocative natural scenes. Using eye-tracking, we recorded eye-movements of 25 HD participants (advanced pre-symptomatic and early symptomatic) and 25 age-matched unaffected control participants during a picture viewing task. Participants viewed pictures of natural scenes associated with different emotions: anger, fear, disgust, happiness, or neutral, and evaluated those pictures on a valence rating scale. Individuals with HD displayed abnormal visual scanning patterns, but did not differ from controls with respect to their valence ratings. Specifically, compared to controls, HD participants spent less time fixating on the pictures and made longer scan paths. This finding highlights the importance of taking visual scanning behavior into account when investigating emotion processing in HD. The visual scanning patterns displayed by HD participants could reflect a heightened, but possibly unfocussed, search for information, and might be linked to attentional deficits or to altered subjective emotional experiences in HD. Another possibility is that HD participants may have found it more difficult than controls to evaluate the emotional valence of the scenes, and the heightened search for information was employed as a compensatory strategy.

## Introduction

Huntington’s disease (HD) is an autosomal dominant neurodegenerative movement disorder. Neural loss originates in the basal ganglia, and spreads progressively to a wide range of subcortical and cortical regions (Vonsattel et al., 1985, 2008; Aylward et al., 2004). HD is characterized by motor impairments, cognitive decline, and psychiatric symptoms (Craufurd et al., 2001; Paulsen and Conybeare, 2005; Van Duijn et al., 2007). The gene mutation that causes HD was identified in 1993, and a person’s genetic status can now be determined before the diagnosis of HD (The Huntington’s Disease Collaborative Research Group, 1993). The symptoms of disease most typically do not manifest until middle adulthood (Folstein, 1989).

A large body of research indicates that people with HD have deficits in the processing of emotional stimuli, even prior to the onset of motor symptoms, i.e., in the pre-symptomatic phase of disease (c.f., Sprengelmeyer et al., 1996, 2006; Mitchell et al., 2005; Bora et al., 2016). These deficits are typically investigated by presenting participants with emotion-related stimuli and asking them to identify or evaluate the emotional content. The most frequently reported deficit is an impaired ability to *identify emotions* from pictures of actors posing with emotional facial expressions (e.g., Sprengelmeyer et al., 1996; Johnson et al., 2007; Labuschagne et al., 2013; Bora et al., 2016). Furthermore, many studies have found alterations in the self-reported *experience* of emotion in response to emotionally evocative stimuli (c.f., Mitchell et al., 2005; Paradiso et al., 2008; Eddy et al., 2011; Ille et al., 2011a; De Tommaso et al., 2013).

Interestingly, findings regarding the self-reported experience of emotion vary depending on stimulus type. Studies using smells or verbal descriptions to induce an experience indicated diminished subjective experiences of disgust or fear in HD (Mitchell et al., 2005; Hayes et al., 2007; Eddy et al., 2011). In contrast, when pictures of emotionally evocative scenes were used most studies found exaggerated emotional experiences in HD. Specifically, findings include exaggerated experiences of specific emotions, such as anger, fear, disgust, or happiness (Paradiso et al., 2008; Eddy et al., 2011; Ille et al., 2011a), and exaggerated experiences of arousal or valence (Paradiso et al., 2008; De Tommaso et al., 2013). Findings, however, also include a reduced experience of fear in HD (Eddy et al., 2011), as well as a lack of group differences when rating the experience of several emotions, including, disgust, happiness, or fear (Eddy et al., 2011; Ille et al., 2011b). Studies into the neural correlates of processing emotional scenes, using [^15^O]water positron emission tomography (PET; Paradiso et al., 2008), electroencephalography (EEG; De Tommaso et al., 2013) and voxel-based morphometry (VBM; Ille et al., 2011b) showed associations between self-reported experiences and structural and functional changes in widespread brain regions in HD, including frontal-subcortical emotion processing networks. Overall, although emotional experiences appear to be affected in HD, the ambiguous findings from self-report studies do not allow for clear conclusions about whether experiences are generally diminished or exaggerated, which emotional states are affected, and whether this depends on the type of stimulus. A possible explanation for the ambiguous findings maybe that self-report is unreliable in HD due to cognitive impairment (Duff et al., 2010; Paulsen, 2011), lack of insight (Ho et al., 2006), and alexithymia (Eddy and Rickards, 2015). Additionally, the difference in self-report findings for visual compared to non-visual stimuli suggests that alterations in visual processing may contribute to the altered evaluations of visual stimuli.

The purpose of the present study was to provide new insights into the processing of emotional stimuli in HD by addressing the question of whether people with HD show abnormalities in parameters characterizing global visual scanning behavior when viewing emotionally evocative natural scenes, compared to healthy control participants, as measured using eye-tracking. Visual scanning behavior was investigated for two reasons. Firstly, altered cognitive, emotional or motivational processes that take place during emotion processing in individuals with HD may influence visual scanning behavior and, therefore, observed alterations in scanning behavior in HD may be indicative of these processes. Considering the ambiguous self-report findings and the limits of asking individuals with HD to report on their own experience, the examination of eye-tracking parameters may add to a better understanding of how people with HD process emotionally evocative stimuli. Secondly, the altered evaluations of visual stimuli in HD may be caused by abnormal visual scanning. Specifically, visual scanning behavior affects the sensory information available for further processing during visual perception, and could therefore contribute to abnormal evaluations.

Eye-tracking enables us to identify parameters characterizing global visual scanning behavior. These parameters are related to the fixations and saccades made on a visual stimulus, and allow specific inferences regarding the processes taking place in the observer during visual perception. In particular, longer fixation durations typically indicate a deeper and more extensive processing of visual stimuli (e.g., Irwin, 2004) and saccades determine the extent of information intake. Visual scanning parameters are influenced by the perceptual properties and spatial composition of an image (e.g., Peters et al., 2005; Unema et al., 2005; Bradley et al., 2011), by task relevance (e.g., Henderson et al., 1999), and by the emotion experienced by the observer (e.g., Castelhano et al., 2009; Bradley et al., 2011; Kaspar et al., 2013). With respect to the emotional state of the observer, Bradley et al. (2011) found longer scan paths and more fixations for emotionally engaging scenes, compared to neutral scenes, from the International Affective Picture System (IAPS; Lang et al., 1997), which they interpreted as reflecting enhanced information seeking.

The literature on HD offers several findings suggesting that the visual scanning of emotional scenes may be altered. Firstly, several previous eye-tracking studies in HD found alterations in visual scanning behavior during the completion of cognitive tasks, including tasks measuring attention (Fielding et al., 2006), inhibitory control (Henderson et al., 2011), or processing speed and memory (Blekher et al., 2009). Secondly, certain alterations in visual processing appear to occur as a correlate of processing emotional stimuli, which may be related to alterations in scanning behavior. That is, investigations into the neural correlates of facial emotion recognition in HD, using EEG (Croft et al., 2014), fMRI (Dogan et al., 2013), and MRI (Harrington et al., 2014), indicated an association between altered visual processing and emotion recognition. Nevertheless, a single study in HD that measured eye-movements during emotion processing did not find evidence for altered visual scanning (Van Asselen et al., 2012). This study, however, took a very different approach, compared to the approach in our study, by examining the visual scanning of faces, rather than scenes, and by measuring the scanning of specific regions of interest, rather than measuring global scanning patterns. Specifically, the study looked at the number of fixations and fixation durations for the eye, nose, and mouth regions, and found no differences between the HD and control groups (Van Asselen et al., 2012). Van Asselen et al. (2012) suggest that this lack of group differences in visual scanning indicates that impaired facial emotion recognition in HD may occur due to a high-level emotional mechanism, rather than due to altered visual scanning. In contrast to the visual scanning of facial features, no research in HD to date has examined parameters characterizing global visual scanning behavior for emotional scenes.

In addition to research in HD, findings in Parkinson’s disease (PD) also raise the possibility that people with HD may show altered visual scanning of emotional scenes. PD is a motor disorder that shares similarities with HD in terms the affected neural circuitry and is associated with deficits in emotion processing similar to those present in HD (Gray and Tickle-Degnen, 2010; Kordsachia et al., 2016). Although people with PD visually scan faces similarly to healthy control participants, in terms of fixation characteristics and locations (Clark et al., 2010), global viewing behavior for emotional scenes has been found to be altered in PD (Dietz et al., 2011). Specifically, compared to controls, PD participants showed abnormalities in quantitative parameters characterizing global viewing activity when passively viewing emotionally evocative natural scenes from the IAPS, including fewer fixations and shorter scan paths, in particular for pleasant pictures (Dietz et al., 2011). This scanning pattern displayed by people with PD is consistent with reduced information seeking and the authors suggest that this behavior may result from a lack of motivation, in line with the frequent occurrence of apathy in PD. The findings in PD of unaffected scanning locations when viewing emotional faces, but abnormal global viewing activity for emotional scenes, considered together with known overlaps in neural circuitries affected and emotional processing abnormalities shared across PD and HD, highlights the possibility that parameters characterizing the global scanning of scenes may also be altered in HD. Thus, although the only study that has looked at scanning of facial features in HD did not find a deficit in visual scanning (Van Asselen et al., 2012), we wished to examine the global visual scanning of scenes to see whether we would find alterations in HD.

In the present study, we recorded eye-movements and examined global visual scanning behavior during a picture viewing task that involved rating the emotional valence of natural scenes, and compared individuals with the gene-expansion for HD to control participants. We were interested in fixations as an indication of the depth of information processing. Therefore, we examined the portion of viewing time spent fixating on a visual scene, which is a function of the average duration and the number of fixations. We were also interested in saccades, as an indication of the extent of picture exploration. Specifically, we examined the scan path length, which is a function of the number of saccades and the average saccade length and can be interpreted as reflecting the degree of information seeking. Based on findings in HD showing deficits in the processing of emotional stimuli and abnormal visual scanning during cognitive tasks, and findings of altered visual scanning of emotionally evocative scenes in PD, we hypothesized that individuals with HD would show abnormalities in the duration and number of fixations, and with respect to length and number of saccades made when exploring the scenes. The complex array of previous findings related to the processing of emotional stimuli in HD, and the fact that eye-tracking research is sparse, made it difficult to make more specific predictions regarding how scanning patterns in HD may be altered. One possible prediction would be that altered processing of the emotional content of scenes in HD leads to abnormal scanning behavior. For example, exaggerated emotional experiences would be expected to result in long scan paths and frequent fixations, consistent with an enhanced search for information. Another possibility is that people with HD show altered visual scanning behavior due to disease-related changes in motivation or in motor and cognitive functioning, which could be reflected in self-report evaluations of emotional scenes due to different availability of visual information secondary to the scanning alternations. For example, people with HD, similar to people with PD, may show fewer fixations and longer scan paths than controls, maybe due to a lack of motivation. By contrast, a lack of differences between the HD and control groups in visual scanning would suggest that abnormal self-report evaluations of emotional scenes in HD are unlikely to result from altered visual scanning.

## Materials and Methods

### Participants

Twenty-five participants with the gene-expansion for HD and 25 healthy control participants took part in this study. Participants were recruited through local advertisement and participant databases. HD participants included pre-symptomatic and early symptomatic individuals that were genetically confirmed (CAG repeat length ≥ 39), expect for one symptomatic participant whose CAG status was indirectly verified by having a genetically confirmed offspring. The HD participants were included based on their Disease Burden Score (DBS; Penney et al., 1997; Tabrizi et al., 2009) and Total Functioning Capacity (TFC) from the Unified Huntington’s Disease Rating Scale (UHDRS; Huntington Study Group, 1996). The DBS is an estimate of an individual’s lifetime exposure to mutant huntingtin at any age, before and after diagnosis (formula: age × [CAG–35.5]), and inclusion as a pre-symptomatic participant required a DBS of ≥270, which excludes individuals that are expected to be more than 10 years from the clinical diagnosis. The TFC indicates the stage of functional deficits in HD patients, with higher scores representing better function. Inclusion as a symptomatic participant required a TFC of ≥7; thus, symptomatic HD participants included individuals with minimal clinical impairment (TFC closer to the maximum score of 13) and moderate clinical impairment (TFC scores of 7–8). We collected further demographic and health information to characterize the participant sample, including: age, years of education, handedness, depressive symptoms using the Quick Inventory of Depressive Symptomatology (QIDS-SR16; Rush et al., 2003), and apathy using the Apathy scale (AS; Starkstein et al., 1992), as well as additional disease related information in the HD group, including the UHDRS motor score, diagnosis, and medication (**Table 1**). *T*-test showed that groups did not differ significantly with respect to age and education [age: *t*(48) = 0.08, *p* = 0.94; education: *t*(48) = 1.31, *p* = 0.20]. All study procedures were approved by the Monash University Human Research Ethics Committee (MUHREC) and written informed consent in accordance with the Declaration of Helsinki was obtained from all participants.

**Table 1:** Characteristics of study participants.

	HD group				Control group			
	Mean	*SD*	Min–Max	*N*	Mean	*SD*	Min–Max	*N*
Age (years)	49.4	8.8	36–67	25	49.6	8.9	35–66	25
Gender (F:M)	14:11	−	−	25	15:10	−	−	25
Education (years)	14.8	3.2	10–22	25	15.8	2.4	11–20	25
Handedness (R:L)	23:2	−	−	25	22:3	−	−	25
AS	13.6	5.7	4–33	25	11.4	7.2	0–30	25
QIDS 16 SR	5.8	4	1–7	25	3.6	2	0–16	25
CAG repeat length	43	1.5	40–47	24	−	−	−	−
DBS	361	75.6	270–517	24	−	−	−	−
TFC score	11	2.3	7–13	25	−	−	−	−
UHDRS motor score	8.3	9.2	0–30	24	−	−	−	−
Diagnosed with symptomatic HD (Y:N)	12:13	−	−	25	−	−	−	−
No HD related medication	*60%*							−
Medicated for depression	*32% (24% SSRI; 4% SNRI; 4% NaSSA and SSRI)*							−
Medicated for chorea	*16% (8% Haloperidol; 4% Risperidone; 4% Clonazepam)*							−

*AS, The Apathy Scale; QIDS 16 SR, Quick Inventory of Depressive Symptomatology 16 SR; DBS, Disease Burden Score; TFC, Total Functioning Capacity; UHDRS, Unified Huntington’s Disease Rating Scale; SSRI, selective serotonin reuptake inhibitor; SNRI, serotonin–norepinephrine reuptake inhibitor; NaSSA, noradrenergic and specific serotonergic antidepressant; N is 24 for variables where values were not available for all participants.*

### Picture Viewing Task

**FIGURE 1:**
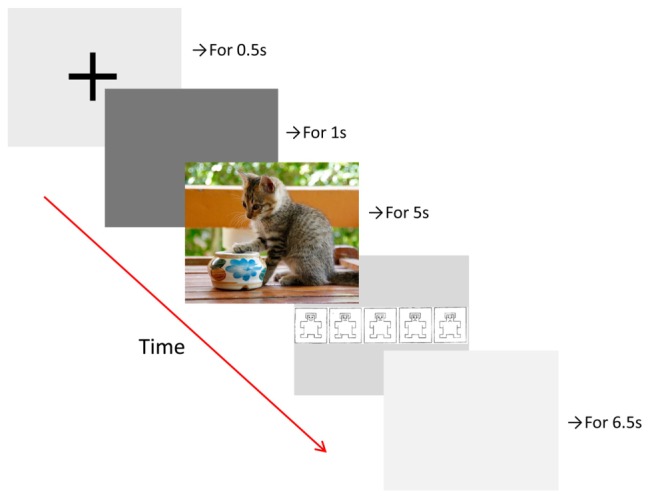
**Time course of trials in the picture viewing task with example picture (not from the IAPS)**.

During the picture viewing task, which was programmed using Presentation software (Neurobehavioral Systems, Inc., Berkeley, CA, USA), participants rested their head on a chin rest, at a distance of 80 cm from the display monitor (22-inch widescreen LCD monitor). The run-time of the task was around 30 min. Pictures of natural scenes (720 pixels × 576 pixels) were presented on a gray background in five randomized blocks (of 10 randomized stimuli), each associated with one of five emotions: anger, fear, disgust, happiness, and neutral. Thus, we used a total of 50 different images, including 10 images per emotion. Each trial consisted of the presentation of a fixation cross (for 0.5 s), a baseline screen (for 1 s), the scene stimulus (for 5 s), and finally a valence rating scale, which was shown until the participant responded via button press (**Figure 1**). The valence rating scale was a five-point Likert scale, ranging from −2 (very negative) to 2 (very positive), that used a pictorial assessment technique, the self-assessment manikin (SAM; Bradley and Lang, 1994). Each trial was followed by an inter-trial interval of 6.5 s, and blocks were separated by breaks that lasted until the participant felt ready to continue. Participants were instructed to sit still, watch the pictures, pay attention to the emotional content, and indicate whether they saw a positive or negative scene using the rating scale. They were also advised that there are no right or wrong answers, and to make a quick and intuitive decision.

The images showed natural scenes involving objects, animals, landscapes, or interactions between people. We purposefully did not include any pictures showing close-ups of human faces since we were interested in the processing of scenes, rather than in facial emotion recognition. Natural scenes are unlikely to ever evoke only one singular emotion in the observer, yet we made an effort to select pictures for each emotion condition that would evoke the particular emotion predominantly. Most stimuli were taken from the IAPS (Lang et al., 1997), and were selected with the help of rating data for specific emotions published by Libkuman et al. (2007). However, anger pictures were mostly taken from the internet, because the IAPS includes few pictures eliciting predominantly anger. Anger pictures included, for example, acts of violence against animals, fear pictures included, for example, dangerous animals, disgust pictures included, for example, dirty toilets, happy pictures included, for example, pictures of baby animals, and neutral scenes were pictures of everyday objects.^1^

Across the emotional conditions, we did not control for stimulus properties, such as brightness and spatial composition. Spatial composition is particularly relevant in the context of this study, as differences in the amount and distribution of details naturally influence scanning patterns, such as scan path length. For this reason, we did not have research questions related to differences between the emotions but presented some eye-tracking variables for each emotion individually, as well as combined, for illustrations purposes.

### Eye-Movement Recording and Processing

An EyeLink 1000 system (SR Research, Ottawa, ON, Canada) was used to record participants’ eye-movements binocularly at a sampling rate of 500 Hz. We calibrated and validated the eye-tracker at the start of the picture viewing task and in-between blocks. The eye-tracker automatically detected saccades based on the three default measures: amplitude of at least 0.1° visual angle, with a velocity of at least 30°/s, and with an acceleration of at least 8000°/s. Fixations were defined as periods without saccades. We analyzed the eye-tracking data for the right eye using the EyeLink Data Viewer (SR Research, Ottawa, ON, Canada). We excluded all trials with less than 50% screen viewing time, assuming that trials with strongly reduced screen viewing time, for example due to distraction, might be associated with abnormal viewing behavior (less than 10% of trails). For the saccade analysis, we excluded saccades that led to locations beyond the screen boundary, as those saccades are not part of the picture exploration.

For each trial, we extracted three fixation and three saccade parameters. Fixation parameters included: (1) the total fixation ratio (calculated as the total fixation duration divided by the trial viewing time, where trial viewing time was defined as the total trial time minus the time not spent viewing, for example due to blinks), which is a function of (2) the average fixation duration, and (3) the number of fixations. Saccade parameters included: (1) the scan path length in degree of visual angle (calculated as the sum of all saccade lengths), which consists of (2) the average saccade length in degree of visual angle, and (3) the number of saccades.

## Statistical Analysis and Results

We used SPSS for Windows (IBM) to conduct statistical analyses. We analyzed the eye-tracking and rating data using analyses of variances (ANOVAs) and *t*-tests. In each ANOVA, we used the Huynh–Feld correction if the sphericity assumption was violated, and Bonferroni corrections for *post hoc* comparisons. Effect sizes are reported as $\eta_{p}^{2}$ for ANOVAs and Cohen’*d* for *t*-tests.

### Group Comparisons on the Duration and Number of Fixations

**FIGURE 2:**
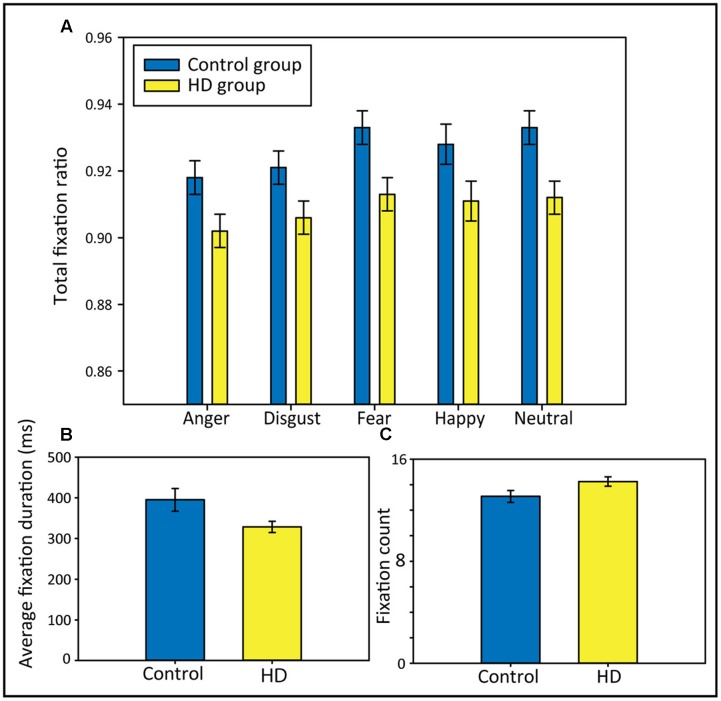
**Total fixation ratios separately for each emotional condition (A)**, average fixation durations in ms **(B)**, and fixation counts **(C)** in both participant groups. Error bars indicate standard error of the mean.

To answer the question of whether HD and control participants differ in the portion of viewing time spent fixating on the scene stimuli, and to test whether possible group differences would differ between the emotional conditions, we analyzed the total fixation ratio using 2 × 5 repeated-measures ANOVAs with the factors Group (HD vs. control) and Emotion (anger, disgust, fear, happy, and neutral). We found that compared to control participants, the HD participants spent a significantly lower portion of viewing time fixating (**Figure 2A**), evident from a significant main effect of *Group*, *F*(1,48) = 6.96, *p* < 0.011, $\eta_{p}^{2}$ = 0.13. There was also a significant main effect of *Emotion*, Huynh–Feldt corrected: *F*(3,145.6) = 18.12, *p* < 0.001, $\eta_{p}^{2}$ = 0.27, where *post hoc* tests revealed significantly lower total fixation ratios for anger and disgust compared to fear, happy and neutral (all *p*-values < 0.05). There was no significant interaction, indicating that the lower total fixation ratio in the HD group occurred independent of the emotional picture content.

To further explain group differences in the total fixation ratio, we calculated *t*-tests comparing both groups on their average fixation durations and their fixation counts (across all five emotional conditions). Results showed that the lower total fixation ratio in the HD group appeared to be a result of shorter average fixation durations, rather than of a lower number of fixations; the *t*-tests comparing the groups regarding their average fixation durations and their fixation counts revealed that the HD participants made significantly shorter average fixations, *t*(48) = 2.15, *p* = 0.037, *d* = 0.61 (**Figure 2B**). Fixation counts did not differ significantly between groups, but HD participants showed a trend toward a higher fixation count than controls, *t*(48) = −1.96, *p* = 0.06, *d* = 0.56 (**Figure 2C**).

### Group Comparisons on the Length and Number of Saccade

**FIGURE 3:**
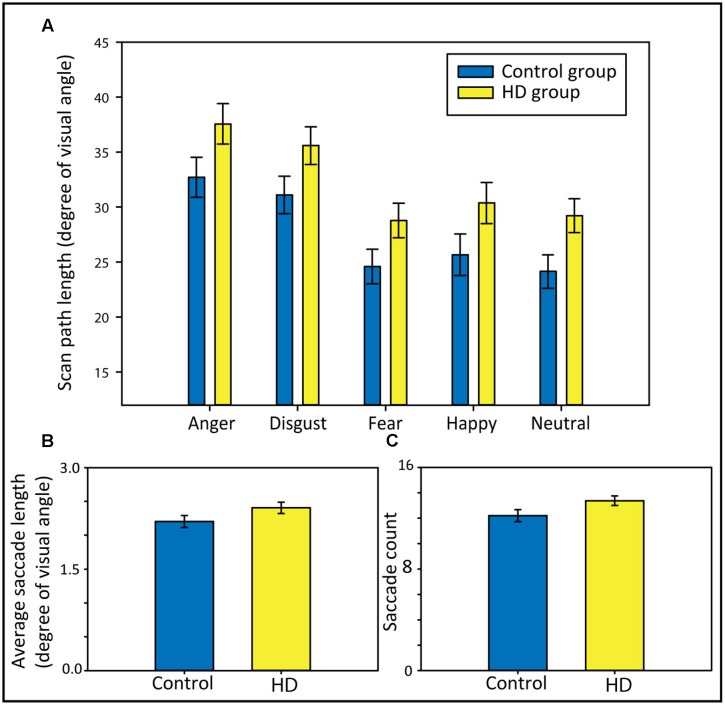
**Scan paths length in degree of visual angle separately for each emotional condition (A)**, average saccade lengths in degree of visual angle **(B)**, and saccade counts **(C)** in both participant groups. Error bars indicate standard error of the mean.

To answer the question of whether HD and control participants show differences in scan path length and to test for possible interactions with the emotional condition, we analyzed the scan path length using a 2 × 5 repeated-measures ANOVA with the factors Group (HD vs. control) and Emotion (anger, disgust, fear, happy, and neutral). We found that HD participants made longer scan paths compared to control participants (**Figure 3A**), evident from a significant main effect of *Group*, *F*(1,48) = 4.64, *p* = 0.04, $\eta_{p}^{2}$ = 0.09. We also found a significant main effect of *Emotion*, Huynh–Feldt corrected, *F*(3.6,171) = 42.81, *p* < 0.001, $\eta_{p}^{2}$ = 0.47, and *post hoc* tests revealed significantly longer scan paths for anger and disgust compared to fear, happy, and neutral (all *p*-values < 0.001). The ANOVA did not show a significant interaction, suggesting that the longer scan paths in the HD group occurred similarly across the different emotional contents.

To further explain group differences in the total scan path length, we calculated *t*-tests comparing both groups regarding their average saccade lengths and their saccade counts (across all five emotional conditions). The longer scan path length in the HD group appeared to result from a combination of somewhat longer (**Figure 3B**) and more frequent saccades (**Figure 3C**). Although neither the group comparison for the average saccade length nor for the saccade count reached statistical significance [*t*(48) = −1.67, *p* = 0.10, *d* = 0.56, and *t*(48) = −1.96, *p* = 0.056, *d* = 0.47, respectively], both showed trends toward longer and more frequent saccades in HD.

### Group Comparisons on Valence Ratings

**FIGURE 4:**
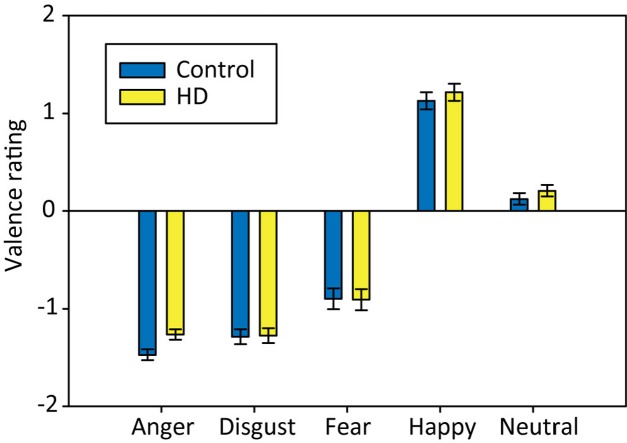
**Mean valence ratings for pictures associated with each emotional condition in both participant groups.** Error bars indicate standard error of the mean.

We analyzed the valence ratings using a 2 × 5 repeated-measures ANOVA with the factors Group (HD vs. control) and Emotion (anger, disgust, fear, happy, and neutral). The results suggested that our stimuli were perceived in the expected way, without differences between HD and control participants (**Figure 4**). Anger and disgust stimuli were rated as very negative. Fear pictures were rated as slightly less negative, possibly due to the choice of scenes, often involving dangerous animals that are likely to be seen as a part of nature and not as inherently negative, or maybe because ‘artificial’ fear stimuli are not able to evoke fear in the same way as real threats, whereas anger and disgust pictures are more suited to evoke an emotional experience. Happy pictures were rated as very positive and neutral pictures as slightly positive (but close to zero). Statistically, the 2 × 5 ANOVA showed a significant main effect of *Emotion*, Huynh–Feldt corrected: *F*(3.4,163.6) = 401.09, *p* < 0.001, $\eta_{p}^{2}$ = 0.89, with *post hoc* tests showing significant differences between all emotional conditions, except for disgust and anger (all *p*-values < 0.01). There were no significant effects of *Group* or *Group* × *Emotion*.

## Discussion

Consistent with our hypothesis of abnormal fixation and saccade behavior during emotion processing in HD, we found that individuals with HD, compared to controls, spent a lower portion of picture viewing time fixating and made longer scan paths. These group differences occurred independent of the particular emotional content of the stimuli. The shorter fixation time was a result of shorter average fixation durations, rather than of a lower number of fixations. HD participants tended to make more fixations than controls, although the group difference was not statistically significant. The longer scan paths appeared to result from a combination of saccades that were somewhat more frequent and slightly longer; although group differences in these two parameters did not reach statistical significance.

Despite the altered visual scanning behavior in HD, we did not find group differences in the valence ratings of the stimuli, indicating that the abnormal scanning patterns did not affect the HD participants’ ability to extract the information necessary for performing those ratings, and that the participants in both groups experienced the valences of the stimuli similarly. A number of previous studies, however, have found altered self-reported emotional experiences in response to visual stimuli in individuals with HD (e.g., Paradiso et al., 2008; Eddy et al., 2011; Ille et al., 2011a). Abnormal visual scanning behavior in HD may have contributed to these previous findings. A possible explanation for the lack of self-report group differences in the present study could be that we used a very basic five-point valence rating scale, rather than more complex ratings for individual emotional states, such as anger, fear, etc. Therefore, our ratings may not have been sensitive enough to detect group differences in emotional experiences between HD and control participants. Importantly, our data imply that future investigations of the processing of emotional visual stimuli, whether they examine self-report or neural correlates of emotional experiences, should consider the influence of scanning behavior.

### Interpretation of Abnormal Scanning Patterns in HD

The visual scanning behavior we observed in the HD participants could reflect a heightened search for information, considering the long scan paths. Considering the short fixations, our findings may also indicate a shallow depth of information processing, i.e., unfocussed processing. The longer scan paths with shorter and somewhat more frequent fixations in our HD sample contrast with the shorter scan paths and fewer fixations that have been observed in PD patients compared to controls, who were studied during passive viewing of IAPS pictures (Dietz et al., 2011). In contrast to a heightened search for information, the scanning behavior displayed by the PD patients is consistent with a low level of picture exploration, and was interpreted as unmotivated or apathetic. Apart from disparities between HD and PD, differences in task requirements between the PD study and our HD study (i.e., free viewing vs. valence rating) may also explain the different findings between the studies, as task instructions can alter eye-movements to meet specific goals (e.g., Einhauser et al., 2008). Specifically, in our study, the requirement to evaluate the stimuli might have had a motivational effect that overwrote a disease-related tendency to make fewer fixations and shorter scan paths. Future research should directly compare HD and PD groups with similar characteristics in terms of disease severity and progression under the same tasks requirements, to examine whether a true disparity exists between HD and PD, or whether both diseases are in fact associated with a reduced search for information under free viewing conditions and a heightened search for information when asked to evaluate the emotional content of visual scenes.

A number of alternative explanations may account for the visual scanning patterns displayed by the HD participants in the current study. Some of these explanations relate to general deficits in HD and are not specific to emotion processing, whereas others are emotion-specific. Firstly, general physiological alterations in HD may play a role, considering that oculomotor abnormalities occur in HD, in particular slowed saccades and delayed saccade initiation (Lasker and Zee, 1997; Hicks et al., 2008; Peltsch et al., 2008). Although these alterations appear to be unlikely to result in longer scan paths and shorter fixations, we cannot exclude the possibility that physiological alterations may be relevant. Another explanation for our findings, that is not emotion-specific, is that the short fixations displayed by HD participants could be related to a general difficulty in focusing attention on specific task-relevant elements of visual scenes, consistent with deficits in general cognitive functioning and attention (Sprengelmeyer et al., 1995; Paulsen and Conybeare, 2005; Fielding et al., 2006).

Alternatively, an explanation for our findings that is specific to emotion processing, could be an exaggerated emotional experience, given that healthy individuals show longer scan paths and more frequent fixations for emotionally engaging IAPS scenes (Bradley et al., 2011). Intriguingly, this is consistent with previous self-report studies suggesting exaggerated emotional experiences in response to emotionally evocative visual scenes in HD (e.g., Paradiso et al., 2008; Ille et al., 2011a). This interpretation, however, is not supported by the valence ratings in our study, which did not suggest exaggerated emotional experience. Additionally, other studies have found a diminished, rather than exaggerated, experience of certain emotions in HD, especially when using non-visual stimuli (Mitchell et al., 2005; Hayes et al., 2007; Eddy et al., 2011). Therefore, future research is needed to clarify whether emotional experience is indeed exaggerated in HD, and which experiences are affected.

Finally, another emotion-related explanation for our observation of longer scan paths in HD participants, compared to controls, could be that the HD participants had difficulties evaluating the emotional valence of the stimuli, and a heightened search of information was employed in an attempt to compensate for these difficulties. Although it is reasonable to assume that cognitive impairment may have made it more difficult to evaluate the emotion-related elements of visual scenes, previous research suggests that the conceptual understanding of emotions remains relatively intact in HD, even in symptomatic individuals (Hayes et al., 2007; Snowden et al., 2008; Calder et al., 2010). Instead, people with HD may have less capability for interocepting their own emotional experience, which is consistent with studies in HD suggesting alexithymia (Eddy and Rickards, 2015) and a lack of insight (Ho et al., 2006). Difficulties evaluating emotional stimuli in HD are in line with the mixed findings from previous research related to experience-ratings in HD (e.g., Mitchell et al., 2005; Paradiso et al., 2008; Ille et al., 2011a), and might explain the inconsistencies regarding the direction (i.e., exaggerated or diminished) of alterations. To gain a better understanding of the causes for the scanning patterns displayed by HD participants, and, in particular, to understand whether the observed effects are specific to emotion processing, future research should compare the scanning of natural scenes during the evaluation of emotional and non-emotional stimulus properties.

### Limitations and Conclusion

An important limitation of this study is the small participant sample, which is particularly relevant because of the variability in clinical phenotypes of HD. Additionally, future studies in HD could go beyond basic valence ratings as an indication of emotional experience, which may not be sensitive enough to detect group differences, thus potentially adding a richer set of data about the association of visual scanning behavior in HD with self-reported emotional experience.

Overall, this study found that HD participants displayed abnormalities in fixation and saccade behavior when processing the emotional content of natural scenes, which are consistent with enhanced, but possibly unfocussed, information seeking. These findings support previous self-report and neurophysiological studies suggesting altered processing of emotional visual stimuli in HD, and add an interesting element to understanding how emotion processing may be affected. This is the first investigation of visual scanning behavior for emotional visual scenes in HD, and although the results do not point to a singular explanation for the observed scanning behavior, they open up interesting questions for future research. We discussed a number of alternative explanations for the scanning patterns displayed by HD participants, including explanations that are not specific to emotion processing, such as physiological alterations and attentional problems, as well as emotion-specific explanations, such as an exaggerated emotional experience and difficulties evaluating emotional valence. Whether or not the observed effects are emotion-specific, our findings highlight the importance of taking visual scanning into account when investigating the processing of emotional scenes in HD, as altered scanning behavior may affect stimuli evaluations as well as their neural correlates.

## Author Contributions

CK conceptualized the study, collected, processed and analyzed the data, and prepared the manuscript. IL and JS contributed to the conceptualization of the study and critically revised and edited the manuscript.

## Conflict of Interest Statement

The authors declare that the research was conducted in the absence of any commercial or financial relationships that could be construed as a potential conflict of interest.

## Footnotes

1 IAPS pictures for happy were: 1460, 1604, 1610, 1630, 1710, 1920, 2388, 5001, 5779, 7330; IAPS pictures for disgust were: 7360, 7380, 9008, 9291, 9300, 9301, 9325, 9342, 9373, 9830; IAPS pictures for fear were: 1022, 1052, 1201, 1300, 1303, 1930, 1931, 5972, 6230, 7640; IAPS pictures for neutral were: 7004, 7009, 7010, 7012, 7021, 7025, 7052, 7081, 7179, 7235; Anger pictures included IAPS picture 2751, as well as depictions of acts of violence against animals or children, irresponsible behavior, such as littering or smoking in a car with children, and frustrating situations, such as a last minute flight cancelation at an airport. Anger pictures from the internet where chosen through a small pilot study where lab members were asked to categorize preselected images.
